# Railroad Traveling

**Published:** 1873-05

**Authors:** 


### RAILROAD TRAVELING.

THE safest part of the car is about the middle, in a seat next the aisle.

If the feet are inclined to be cold, carry with you an air-cushion, or place your shawl or great coat under your feet; this not only keeps the feet warm, but breaks that most unpleasant jarring which is communicated from the floor.

Eat nothing whatever between the regular meal times. If any meal must be taken on the way, supply yourself with two slices of bread and some meat between. If you must eat something before you end your journey, what you want to eat let it be all eaten at the same time ; to be eating some little thing every hour or two is most pernicious, especially to young children.

Always eat a good warm meal, leisurely, before taking a railway journey ; on an emergency this will last you twelve hours, with the aid of a plain sandwich. Before you get into the cars, see that every article of luggage you have has been put in, and the checks deposited in your own hands.

Settle your bills before you take the last meal at your hotel.

Avoid, if possible, starting on a journey sooner than an hour after a regular breakfast ; better reach your journey's end at midnight than to be waked up before your sleep is out and to have to eat in a hurry.

Night travel on steamboats and rail-cars is one of the great vexations of summer travel ; is unhealthy in all its connections, and unfits for the enjoyment of the next day.

Travel is a great leveler. The common sentiment of any company places a man at his real worth, almost by instinct. Clerks, waiters, drivers and bootblacks determine the status of an individual, almost at a glance. Bluster and brag, a loud voice, a petulant tone, an angry complaint, worry and fluster, are translated at once to mean, "You are nobody."

There is a quiet composure, an unostentatious demeanor, and a uniform patience and forbearance in all well-bred persons, which is recognized everywhere as the stamp of good breeding.

Loud talking in a public conveyance, especially when it is evident that the parties either do not care whether they are heard or not, or, as is often the case, prefer to be heard, is the grossest ill-breeding. No person of any innate delicacy could possibly act thus, unless under great forgetfulness—a forgetfulness quite inexcusable.

Nothing more distinctly marks a gentleman than promptitude in vacating his seat for a woman, or in yielding it to gray hairs.

Haste and crowding past others to secure a vacated seat marks the boor; a gentleman stands, looks around to see whether a woman or older person is provided with a seat; or, if he has entered the conveyance and finds one standing, he offers the seat to the one standing longer than himself.

Composure, patience and forbearance mark the gentleman and lady, everywhere.

